# The number of erupted teeth as a risk factor for dental caries in eighteen-month-old children: a cross‑sectional study

**DOI:** 10.1186/s12903-023-03394-0

**Published:** 2023-09-16

**Authors:** Masatoshi Otsugu, Yusuke Mikasa, Maika Kadono, Taro Matsuoka, Katsura Matsunami, Motomi Nakamura, Yuko Ohno, Takafumi Kato, Kazuhiko Nakano

**Affiliations:** 1 Department of Pediatric Dentistry, Osaka University Graduate School of Dentistry, Osaka, Japan; 2 Toyonaka City Public Health Center, Osaka, Japan; 3 Department of Health Sciences, Osaka University Graduate School of Medicine, Osaka, Japan; 4 Department of Oral Physiology, Osaka University Graduate School of Dentistry, Osaka, Japan

**Keywords:** Early childhood caries, Tooth eruption, Birth order, Microbiological test, Breastfeeding

## Abstract

**Background:**

Dental caries is one of the most common chronic diseases worldwide, affecting lifelong as well as children. Therefore, it is important to clarify factors related to early childhood caries (ECC) in a younger population in terms of caries prevention. However, the prevalence of ECC is low in developed countries in the twenty-first century and a large-scale survey is needed to clarify the risk factors. Furthermore, earlier tooth eruption is not taken into consideration in most studies of ECC, even though it may be a factor of ECC. The present study investigated the prevalence and risk factors of dental caries in children aged 18 months in a core city of Japan.

**Methods:**

Findings from a total of 7351 children aged 18 months were analyzed. Anthropometric measurements of height and weight, as well as an oral examination and a microbiological caries-risk test, were performed. Additionally, a structured interview sheet was provided to the parents or guardians. Findings of dental caries at 18 months of age were evaluated using a logistic regression model.

**Results:**

Of the enrolled children, 1.2% had experienced dental caries. Multivariable logistic regression analysis results indicated a significant association with dental caries at 18 months of age for the following factors: second child (OR = 1.78; 95% CI:1.08–2.93, *P* < 0.05), third and later child (OR = 2.08; 95% CI:1.12–3.89, *P* < 0.05), 12 or fewer erupted teeth (OR = 0.47; 95% CI:0.24–0.96, *P* < 0.05), 17 or more erupted teeth (OR = 4.37; 95% CI:1.63–11.7, *P* < 0.01), Cariostat score (+ + +) (OR = 3.99; 95% CI:1.29–12.31, *P* < 0.05), daily eating before bed (OR = 2.62; 95% CI: 1.55–4.45, *P* < 0.001), three or more snacks per day (OR = 2.03; 95% CI:1.15–3.58, *P* < 0.05), and breastfeeding (OR = 3.30; 95% CI:2.00–5.44, *P* < 0.001).

**Conclusions:**

These results suggest that the number of erupted teeth, as well as birth order, eating habits, and breastfeeding, are significant factors in dental caries occurrence at 18 months of age.

## Background

Dental caries is one of the most common chronic and progressive childhood diseases worldwide and can have a negative impact on a child’s growth, general health, and quality of life [1, 2]. It not only affects children; it is a lifelong condition that tracks across adolescence and adulthood [3]. Some epidemiological studies indicate that the lifetime prevalence of dental caries has decreased in recent decades, mainly in developed countries [4, 5]. Early childhood caries (ECC) remains a significant disease of childhood and cause of public health problems [6]. Although dental caries prevalence in primary teeth has decreased in Japan as a result of improved knowledge about its prevention, it still occurs in certain high-risk populations, and the polarization of oral health in children is becoming more apparent [7–10].

Dental caries is a multifactorial disease, encompassing physical, biological, environmental, behavioral, and lifestyle-related factors, such as high levels of cariogenic bacteria, inadequate salivary flow, poor oral hygiene, inappropriate methods of feeding infants, secondhand smoke, and socioeconomic status [11]. Among them, oral bacteria, diet, host, and time are the fundamental factors that directly contribute to dental caries development [11]. However, few studies of ECC adequately take these factors into consideration. In particular, early tooth eruption can be considered as a factor in the development of severe ECC, because of the longer periods of exposure to cariogenic factors such as the colonization of cariogenic bacteria [12]. Nevertheless, few studies have investigated the relationship between tooth eruption and dental caries, and thus there is no definition for early tooth eruption in the eruption stage of primary teeth. Furthermore, the prevalence of severe ECC is low in developed countries in the twenty-first century, and a large-scale survey is needed to clarify the risk factors [10, 13].

In the present study, we investigated the prevalence and risk factors of dental caries in relation to the number of erupted teeth in children aged 18 months in a core city of a developed country, Japan.

## Methods

### Study design

This study was a cross-sectional and complete enumeration study in a city. Data for the study were obtained from all three public health centers in Toyonaka city, a core city to the north of Osaka City, a metropolitan area in western Japan, with a total population of approximately 400,000. In Japan, when children reach 18 months of age, the local municipality performs a physical examination, which includes an oral examination, anthropometric measurements of height and weight, and an interview sheet about the guardian and the child’s health. The public healthcare center mails notices about the examination to families with a child aged 18 months.

### Subjects

The subjects of this study were 8281 18-month-old children (participation rate ≥ 95%) who underwent physical examinations at all three public healthcare centers in Toyonaka City from April 2018 to March 2020. The inclusion criterion was any child who had undergone this clinical examination. The exclusion criterion was any child whose examination data or the interview sheet provided by the parents or guardians were incomplete.

### Clinical examination

In the physical examination, anthropometric measurements of height and weight were recorded. In the oral examination, the number of erupted teeth and carious teeth was assessed visually according to criteria established by the World Health Organization [14]. The oral examination was performed by dentists belonging to Toyonaka Dental Association.

### Microbiological assessment

The microbiological assessment was performed using the Cariostat® (Dentsply Sirona, Tokyo, Japan), which is a colorimetric caries-risk test for measuring the presence of acidogenic microorganisms [15]. Dental plaque was collected from the buccal surfaces of all maxillary teeth using a sterilized cotton swab supplied in the kit. The collected plaque on the swab was put into a test medium in a Cariostat® test tube and incubated at 37 °C for 48 h. The color turns from blue to green and, ultimately, to yellow, indicating increased acid production of the plaque in the sample. The color of the culture medium was compared with the reference color on the color chart supplied. The reference color was scored and classified into four categories, (-), ( +), (+ +), and (+ + +) [15].

### Interview sheet

The structured self-administered interview sheet made by the public healthcare center in Toyonaka city was provided directly to the guardians in the public healthcare center and collected before the physical examination. The interview sheet included the following information: care environment (birth order, commuting to daycare, bedtime) and diet habits (snacking, feeding, eating before bedtime) of the child, and health condition of the guardian (exhausted with parenting, enjoying parenting, anxiety, physical health). Birth order (first, second, third or later), body mass index (BMI; underweight, ideal, overweight), number of erupted teeth (≤ 12, 13–16, 17 ≤), eating before bed (never, weekly, daily), snacking habits per day (never, 1 or 2 times, 3 times or more), feeding habits (weaned, bottle, breast, breast and bottle) and bedtime (before 21:00, 21:00–22:00, after 22:00, unsettled) were categorized as binary variables.

### Statistical analysis

Descriptive analyses included calculation of relative frequencies, absolute frequencies, medians, and interquartile ranges (IQRs). Dental caries prevalence was defined as the frequency of children with at least one carious tooth. The relationship between dental caries prevalence and the number of erupted teeth was analyzed using the Cochran-Armitage test. Additionally, within the range of the number of erupted teeth with dental caries, we performed forward stepwise binary logistic regression with dental caries occurrence at 18 months of age as the dependent variable. All variables with a *P* value lower than 0.05 in the univariate analysis were entered in the regression models as independent variables. The odds ratios (ORs) and their 95% confidence intervals were estimated with regard to risk factors for dental caries occurrence at 18 months of age. The fit of the data to the model was tested using the Hosmer–Lemeshow test and multicollinearity was assessed by using the variance inflation factor (VIF).

For reliable analysis, we required at least 10 events of the primary outcome measure per variable; that is, an estimated, 250 events for 25 variables [16]. Given a prevalence of 4% for dental caries in participants aged 18 months [13], the number of subjects required was estimated at 6250 or more. All data were analyzed using IBM SPSS Statistics version 28.0.1.0® (IBM Japan, Tokyo, Japan) and the level of statistical significance was set at *P* < 0.05.

## Results

**Fig. 1:**
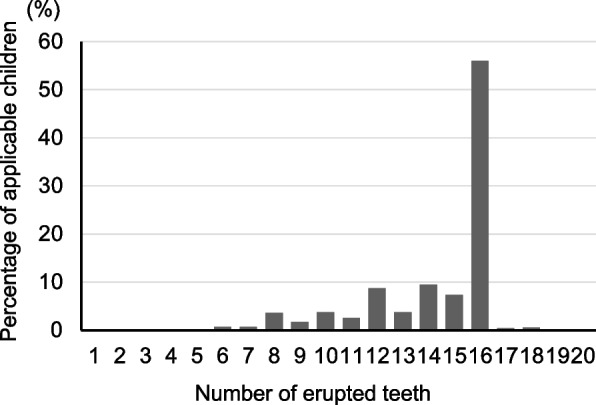
Distribution of the number of erupted teeth

**Fig. 2:**
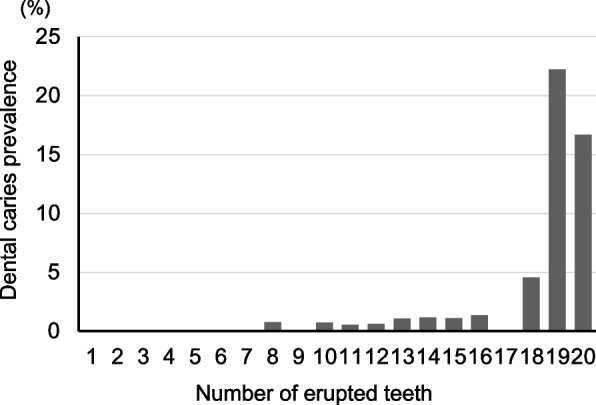
Relationship between dental caries prevalence and the number of erupted teeth (*P* < 0.001: Cochran-Armitage test)

Of the 8281 children, 7351 (88.8%) (3805 boys [51.8%] and 3546 girls [48.2%]) were included. There were no deficiencies in their clinical examination data or the interview sheet answered by parents or guardians. The median height was 79.3 cm (IQR: 77.5–81.2), median weight was 10.3 kg (IQR: 9.6–11.1), and median BMI was 16.0 kg/m^2^ (IQR: 14.9–17.2). Of the 7351 children, 87 (1.2%) had experienced dental caries and 7264 (98.8%) were caries-free. In the 87 children with dental caries, the median number of carious teeth per child was 3 (IQR: 2–4). The number of erupted teeth was 16 (IQR: 14–16) (Fig. 1). Figure 2 shows the relationship between dental caries prevalence and the number of erupted teeth. Dental caries prevalence significantly increased with the number of erupted teeth (*P* < 0.001). Additionally, some of the children with 8 or more teeth had dental caries, but none of the children with 7 or fewer teeth had experienced dental caries. Therefore, further caries risk analyses were performed in children with 8 or more erupted teeth because this was considered to be a caries-sensitive developmental stage.

Of the 7351 children, 7222 (98.2%) (3747 boys [51.9%] and 3475 girls [48.1%]) had 8 or more erupted teeth. The median height was 79.3 cm (IQR: 77.5–81.2), weight was 10.3 kg (IQR: 9.7–11.1), and BMI was 16.0 kg/m^2^ (IQR: 15.0–17.2). Tables 1 and 2 show the results of the univariate analysis of dental caries distribution among the 7222 children with 8 or more erupted teeth at 18 months of age and the risk factors attributed to children and their guardians. The following factors were significantly associated with dental caries occurrence: birth order (second: *P* < 0.01; third or later: *P* < 0.001), number of erupted teeth (≤ 12: *P* < 0.05; ≥ 17: *P* < 0.001), Cariostat score (+ +  + : *P* < 0.001), eating before bed (weekly: *P* < 0.01; daily: *P* < 0.001), snacking (≤ 2 times: *P* < 0.05; ≥ 3 times: *P* < 0.001), feeding habits (bottle feeding: *P* < 0.01; breast feeding: *P* < 0.001; breast and bottle feeding: *P* < 0.01) and bedtime (after 22:00: *P* < 0.01). Commuting to daycare and guardians’ health conditions were not significantly associated with dental caries occurrence. Table 3 shows the results of the binary logistic regression analyses of risk factors associated with dental caries occurrence at 18 months of age. Adjusted by potential confounders, the following factors were significantly associated with dental caries occurrence at 18 months of age: second child (OR = 1.78; 95% CI:1.08–2.93, *P* < 0.05), third or later child (OR = 2.08; 95% CI:1.12–3.89, *P* < 0.05), 12 or fewer erupted teeth (OR = 0.47; 95% CI:0.24–0.96, *P* < 0.05), 17 or more erupted teeth (OR = 4.37; 95% CI:1.63–11.7, *P* < 0.01), Cariostat score (+ + +) (OR = 3.99; 95% CI:1.29–12.31, *P* < 0.05), daily eating before bed (OR = 2.62; 95% CI: 1.55–4.45, *P* < 0.001), snacking three times or more per day (OR = 2.03; 95% CI:1.15–3.58, *P* < 0.05), and breastfeeding (OR = 3.30; 95% CI:2.00–5.44, *P* < 0.001). In the regression model, the Hosmer–Lemeshow test showed a good fit (*P* = 0.664), and the VIF showed the absence of multicollinearity for each independent variable (< 4.0).

**Table 1:** Dental caries distribution and crude odds ratio (OR) among factors in children

Variables		Total *N* (%)	Caries *N* (%)	Crude OR (95% CI)	*P*
(Overall)		7222			
Caries	Yes	87 (1.2)			
	No	7135 (98.8)			
Sex	Male	3747 (51.9)	52 (1.4)		
	Female	3475 (48.1)	35 (1.2)	0.72 (0.47–1.11)	0.139
Birth order	First	3659 (50.7)	28 (0.8)		
	Second	2708 (37.59)	40 (1.5)	1.94 (1.20–3.16)	0.007
	Third or later	855 (11.8)	19 (2.2)	2.95 (1.64–5.30)	< 0.001
Body mass index	Ideal	4559 (63.1)	52 (1.1)		
	Underweight	1181 (16,4)	9 (0.8)	0.67 (0.34–1.36)	0.261
	Overweight	1482 (20.5)	26 (1.8)	1.55 (0.96–2.49)	0.071
Number of erupted teeth	13–16	5632 (78.0)	72 (1.3)		
	≤ 12	1497 (20.7)	9 (0.6)	0.47 (0.23–0.94)	0.032
	≥ 17	93 (1.3)	6 (6.5)	5.32 (2.26–12.6)	< 0.001
Cariostat score	–	313 (4.3)	5 (1.6)		
	+	5957 (82.5)	48 (0.8)	0.50 (0.20–1.27)	0.144
	+ +	847 (11.7)	23 (2.7)	1.72 (0.65–4.56)	0.276
	+ + +	105 (1.5)	11 (10.5)	7.21 (2.44–21.27)	< 0.001
Eating before bed	No	5138 (71.1)	37 (0.7)		
	Weekly	854 (11.8)	15 (1.8)	2.47 (1.35–4.51)	0.003
	Daily	1230 (17.0)	35 (2.8)	4.04 (2.53–6.44)	< 0.001
Snacking	No	5708 (79.0)	50 (0.9)		
	≤ 2 times	876 (12.1)	16 (1.8)	2.11 (1.19–3.71)	0.010
	≥ 3 times	638 (8.8)	21 (3.3)	3.85 (2.30–6.46)	< 0.001
Feeding habit	Weaned	4777 (66.1)	28 (0.6)		
	Bottle feeding	719 (10.0)	11 (1.5)	2.64 (1.31–5.32)	0.007
	Breast feeding	1626 (22.5)	45 (2.8)	4.83 (3.00–7.64)	< 0.001
	Breast + bottle	100 (1.4)	3 (3.0)	5.25 (1.57–17.53)	0.007
Bedtime	Before 21:00	1904 (26.4)	14 (0.7)		
	21:00–22:00	3235 (44.8)	47 (1.2)	1.65 (0.89–3.04)	0.111
	After 22:00	1358 (18.8)	17 (1.7)	2.64 (1.37–5.06)	0.004
	Unsettled	725 (10.0)	9 (2.7)	1.51 (0.63–3.61)	0.358
Daycare	Yes	2460 (34.1)	60 (1.3)		
	No	4762 (65.9)	27 (1.1)	0.87 (0.55–1.37)	0.549

**Table 2:** Dental caries distribution among children and crude odds ratio (OR) among factors in guardians/parents

Variables		Total *N* (%)	Caries *N* (%)	Crude OR (95% CI)	*P*
(Overall)		7222	87 (1.2)		
Exhausted with parenting	Yes	1322 (18.3)	18 (1.4)	0.86 (0.51–1.45)	0.563
	No	5900 (81.7)	69 (1.2)		
Enjoying parenting	Yes	7156 (99.1)	86 (1.2)	1.27 (0.17–9.22)	0.816
	No	66 (0.9)	1 (1.5)		
Anxiety	Yes	1375 (19.0)	18 (1.3)	0.90 (0.53–1.52)	0.693
	No	5847 (81.0)	69 (1.2)		
Physical condition	Healthy	6434 (89.1)	81 (1.3)	0.60 (0.26–1.38)	0.227
	Unhealthy	788 (10.9)	6 (0.8)

**Table 3:** Binary logistic regression analysis with dental caries occurrence as the dependent variable

	Adjusted odds ratio (95% CI)	*P*	Variance inflation factor
Birth order		0.029	1.03
First	Ref		
Second	1.78 (1.08–2.93)	0.023	
Third or later	2.08 (1.12–3.89)	0.021	
Number of erupted teeth		0.001	1.01
13–16	Ref		
≤ 12	0.47 (0.24–0.96)	0.039	
≥ 17	4.37 (1.63–11.7)	0.003	
Cariostat score		< 0.001	1.01
–	Ref		
+	0.42 (0.16–1.07)	0.070	
+ +	1.20 (0.45–3.26)	0.714	
+ + +	3.99 (1.29–12.31)	0.016	
Eating before bed		0.002	1.16
No	Ref		
Weekly	1.79 (0.94–3.41)	0.078	
Daily	2.62 (1.55–4.45)	< 0.001	
Snacking		0.051	1.12
No	Ref		
≤ 2 times	1.30 (0.71–2.39)	0.401	
≥ 3 times	2.03 (1.15–3.58)	0.015	
Feeding habit		< 0.001	1.09
Weaned	Ref		
Bottle feeding	1.30 (0.61–2.77)	0.502	
Breast feeding	3.30 (2.00–5.44)	< 0.001	
Breast + bottle	2.42 (0.63–9.34)	0.198	

Adjusted for birth order, number of erupted teeth, Cariostat score, eating before bed, snacking, feeding habit, and bedtime

## Discussion

In the present study, the prevalence of dental caries in children aged 18 months was 1.2%. Although few studies have been conducted in such a young population, the prevalence was thought to be lower than that in other countries (18 months of age: dmft = 2.8 ± 2.7 [17]; 1 year old: 1.9%, 2 years old: 10.7% [18]). Additionally, the prevalence was slightly lower than that of previous studies in Japan (3.3%–4.0%) [13, 19]. The present results may be indicative of the oral health literacy of this city. However, ECC affects lifelong oral health into adolescence and adulthood [3], and it is problematic that some children have already experienced dental caries at 18 months of age in developed countries in the twenty-first century. Furthermore, these previous studies were small-scale compared with the present study, and large-scale surveys of young children in different regions are needed to clarify the status of ECC in both developed and developing countries [13, 17–19].

Because few studies have been conducted in such a young population, there is also a lack of research about the association between tooth eruption and dental caries [12]. In the present study, a greater number of erupted teeth was a significant factor associated with dental caries at 18 months of age. Early tooth eruption can be an important risk factor for dental caries in young children, and 17 or more erupted teeth in children aged 18 months may be interpreted as early tooth eruption according to the results of the present study. The eruption timing of primary teeth is affected by both genomic factors and environmental factors [20–22]. Although the cause of increased dental caries risk associated with early tooth eruption could not be determined in the present study, our findings suggest an indirect relationship between the vulnerability of early erupting teeth, the early introduction of a mature diet including sugar-containing foods, and the difficulty of maintaining oral hygiene in young children. Longitudinal studies are needed to investigate up to what age earlier tooth eruption affects dental caries risk.

Cariostat® is a microbiological test that evaluates the acidogenic ability from sucrose of the aciduric bacteria in dental plaque, and is a simple test for identifying high-risk patients for dental caries [15]. Microbiological tests in some cross-sectional and longitudinal surveys show strong associations of dental caries with mutans streptococci and lactobacilli [15, 23]. In the present study, a Cariostat score (+ + +) was a significantly high-risk factor for dental caries. The acquisition and colonization of *S. mutans* are considered to occur at approximately 19–31 months after birth [24], and some studies have detected *S. mutans* in the oral cavity of infants younger than 12 months, albeit at low frequency [25]. The results of the present study suggest that bacteriological factors already exert a strong influence at 18 months of age. However, microbiological tests used alone were considered to have limited value in predicting dental caries in a previous study [23]. Nevertheless, previous studies were not complete enumeration studies like the present study and did not include a population as young as 18 months of age. Microbiological tests can be effective in identifying children with high dental caries activity in large populations of children at an age with a low distribution of dental caries.

There are clear benefits of breastfeeding for a child in terms of health promotion and prevention of diseases such as severe lower respiratory infections, asthma, sudden infant death syndrome, and obesity [26]. It is also possible that early breastfeeding may protect against dental caries by delaying the introduction of sugar-containing foods [27]. However, breastfeeding and baby bottle use beyond 12 months, especially if frequent and/or nocturnal, are associated with ECC [27]. In the present study, breastfeeding up to 18 months of age was significantly related to dental caries. However, 97.2% of the children who continued breastfeeding up to 18 months of age had no caries in the present study, although the subsequent caries risk should be analyzed longitudinally. It is thought that breastfeeding is not a determinant factor but a high-risk factor of dental caries and leads to dental caries in combination with sugar consumption rather than directly leading to dental caries [27]. Therefore, rather than the components of breast milk, the habits and behaviors induced by prolonged breastfeeding may combine with sugar consumption, strongly influencing caries risk.

Birth order was a significant environmental factor for dental caries at 18 months of age in the present study. The association between birth order and dental caries has been investigated in recent studies and the present result was consistent with a previous study [28]. Birth order is thought to be related to oral health condition as well as general health condition [29]. Later-born children are likely to have more sibling influences and less parental attention, resulting in less individual care and earlier exposure to sugar-containing foods compared with first-born children [28]. These habitual eating characteristics were consistent with the habit of eating before bed and snacking frequency, both high-risk factors for dental caries in the present study.

In contrast, no significant associations of dental caries with guardians’ health conditions were found in the present study. Although many studies have focused on the association between caries and guardians’ socioeconomic status or education level, few have explored the association of dental caries with guardians’ physical and mental health conditions [1–4, 7, 8, 11, 12, 27]. However, guardians’ socioeconomic status and education level could be related to health-compromising behaviors and lifestyles, and may affect their health condition as a result [18]. Therefore, it may be important for the long-term maintenance of a child’s oral health condition to evaluate the family environment, including the physical and mental health condition of the guardians.

The present study had several limitations. First, detailed daily intake of food and drink, especially those containing sugar, was not investigated. For example, juice intake in infants before the age of 1 year can be a serious ECC risk factor and should be avoided completely [30]. ECC caries risk assessment could have been more thoroughly evaluated by including these items in the interview sheet. Second, dental hygiene habits such as brushing frequency, fluoride use, and dental care patterns were also not investigated; neither were socioeconomic status and guardians’ education level. These factors can depend on guardians and family and may be important factors. Third, dental caries is a chronic disease, and the present cross-sectional study has a certain level of limitation because there may be time discrepancies between dental caries development and risk factors, especially environmental factors. Fourth, the number of erupted teeth and carious teeth were assessed only visually according to criteria established by the World Health Organization, and we did not verify consistency among examiners or the reliability and validity of the interview sheet. Nevertheless, we consider that dental caries distribution at 18 months, despite its low frequency, merits extensive investigation. The fundamental factors directly contributing to caries development, such as oral bacteria, diet, host, and time, were analyzed in a relatively well-balanced manner in the present study.

## Conclusions

Within the limitations of this cross-sectional study, the following conclusions can be drawn:

- 1.2% of children aged 18 months had experienced dental caries in a core city of a developed country, Japan.
- The number of erupted teeth, as well as birth order, eating habits, and breastfeeding, may be significant factors for dental caries occurrence at 18 months of age.
- Bacteriological factors are a significant influence, and microbiological caries-risk tests are effective for detecting a high risk of ECC in children aged 18 months.

## Data Availability

The dataset used and/or analyzed during the current study available from the corresponding author on reasonable request.

## Abbreviation

ECC: Early childhood caries
